# Single Multiple Cross Displacement Amplification for Rapid and Real-Time Detection of Porcine Circovirus 3

**DOI:** 10.3389/fvets.2021.726723

**Published:** 2021-09-03

**Authors:** Zhibiao Bian, Rujian Cai, Zhiyong Jiang, Shuai Song, Yan Li, Pinpin Chu, Kunli Zhang, Dongxia Yang, Hongchao Gou, Chunling Li

**Affiliations:** ^1^Institute of Animal Health, Guangdong Academy of Agricultural Sciences, Guangzhou, China; ^2^Guangdong Provincial Key Laboratory of Livestock Disease Prevention, Guangzhou, China; ^3^Maoming Branch, Guangdong Laboratory for Lingnan Modern Agriculture, Maoming, China; ^4^Scientific Observation and Experiment Station of Veterinary Drugs and Diagnostic Techniques of Guangdong Province, Guangzhou, China

**Keywords:** PCV3, single multiple cross displacement amplification, detection, probe, quantitative

## Abstract

Since 2016, a novel porcine circovirus, PCV3, has been infecting pigs, causing significant economic losses to the pig industry. In recent years, the infection rate of PCV3 has been increasing, and thus rapid and accurate detection methods for PCV3 are essential. In this study, we established a novel probe-based single multiple cross displacement amplification (P-S-MCDA) method for PCV3. The method was termed as P-S-MCDA. The P-S-MCDA uses seven primers to amplify the capsid gene, and the assay can be performed at 60°C for 30 min, greatly shortening the reaction time. The results of P-S-MCDA can not only be monitored in real time through fluorescence signals but also be determined by observing the fluorescence of the reaction tubes using a smartphone-based cassette. This method demonstrated good specificity and the same sensitivity as qPCR, with a minimum detection limit of 10 copies. In 139 clinical samples, the coincidence rate with qPCR was 100%. The P-S-MCDA can be widely applied in PCV3 detection in laboratories or in rural areas.

## Introduction

Porcine circovirus (PCV), one of the smallest known animal viruses, belongs to the family *Circovirus*. Two members of PCV are well-known, namely, PCV1 and PCV2 (1). In 2016, a novel PCV, PCV3, was identified in the USA. Many other countries have also reported the presence of PCV3 in piglets, for example, the UK (2), Germany (3), China (4), Japan (5), Brazil (6), and Russia (7). PCV3 causes a variety of pathological symptoms in piglets and sows such as porcine dermatitis and nephrotic syndrome (PDNS)-like clinical signs, reproductive failure, cardiac pathology, and multi-system inflammation (8). Virus genome detection from the tissues of infected animals has shown that the degree of PCV3 infection in animals gradually increases, eventually infecting almost all tissues and organs (9). Moreover, the virus has spread rapidly among pigs and wild boars. Strikingly, PCV3 was recently found to be epidemic in cattle, mice, deer, and ticks. The ability of PCV3 to undergo cross-species transmission and circulation among a broad range of animals suggests that it may pose a severe threat to other animals (10). This complicates the control measures for PCV3. To date, the virus has caused huge economic losses in the global pig industry (11). Therefore, it is necessary to develop a rapid and accurate detection method to describe and control the epidemiological characteristics of PCV3.

The common diagnostic methods for PCV3 include virus isolation, indirect immunofluorescence testing, ELISA, and nucleic acid detection. Although virus isolation and indirect immunofluorescence testing are standard detection methods, they are complicated and need to be conducted in laboratories with suitable conditions. Hence, nucleic acid detection methods, including PCR, qPCR, and isothermal amplification, are more widely used in PCV3 detection. Previous nucleic acid detection methods for PCV3 include PCR (12), SYBR Green-based qPCR (13), TaqMan-based qPCR (14), loop-mediated isothermal amplification (LAMP) (15), and recombinase polymerase amplification (RPA) (16). Characteristics of qPCR-based methods are high sensitivity and specificity, but the expensive thermal cycler instruments limit their wide application. In contrast, isothermal amplification assays are usually easy to operate, but their less specific properties limit their use in accurate detection.

Recently, a novel isothermal amplification strategy named single multiple cross displacement amplification (S-MCDA) has been developed that not only is more sensitive than LAMP but also can significantly shorten the reaction time (17). To realize the aim of rapid and accurate detection of PCV3, we established a novel probe based on the S-MCDA method, termed as P-S-MCDA, in this study. The P-S-MCDA is not only capable of quantitative analysis of PCV3 in real time but also specific, sensitive, and easy to operate. Moreover, the P-S-MCDA assay results can be visually determined using a small handheld device. When the method was compared with qPCR in analyzing clinical samples, an equal consistency ratio was obtained. Therefore, our P-S-MCDA assay may provide a priority choice for the rapid diagnosis of PCV3.

## Materials and Methods

### Ethics Statement

All animal experiments were reviewed and approved by the ethical and ethics commission (Institute of Animal Health, Guangdong Academy of Agricultural Sciences, China) under license number SYXK (Yue) 2011-0116. Moreover, sample collection in this study was performed in accordance with national and local laws or guidelines.

### Virus, Bacteria, and Cells

As previously described, PCV2 isolate HN6 (GenBank no: KM035762.1), PCV1, pseudorabies virus (PRV) GD-WH strain (GenBank no: KT936468.1), *Haemophilus parasuis* (HPS) serotype 5, *Streptococcus suis* (SS) serotype 2, and *Actinobacillus pleuropneumoniae* (APP) Serovar 1 were preserved in our laboratory. They were first used to extract nucleic acids and then to evaluate the specificity of the P-S-MCDA.

### Animals and Clinical Samples

In 2020–2021, a total of 139 clinical samples from pig farms in Guangdong Province were collected and sent to our laboratory for detection. These clinical samples included blood, lung, kidney, brain, spleen, lymph node, and tonsil. In addition, 15 blood samples were collected from specific-pathogen-free (SPF) pigs (5 months old) that were purchased from the Laboratory Animal Center of Southern Medical University Guangzhou (4). All samples were stored at −80°C until DNA extraction.

### DNA Extraction

All viral DNA for the study was extracted by using a HiPure Viral RNA/DNA Kit (Magen, China) according to the manufacturer's instructions. Bacterial DNA was extracted using the TaKaRa MiniBEST Bacteria Genomic DNA Extraction Kit Ver. 3.0 (Takara, China) according to the manufacturer's protocol. All of the final DNA was stored at −80°C.

### Plasmid Construction

The construction of plasmids was done exactly as previously described (18). The capsid gene used to construct the plasmid was amplified from nucleic acid positive for PCV3, and the products were purified using the Cycle Pure Kit according to the manufacturer's instructions (Omega, USA). The pMDTM19-T Vector (TaKaRa Biotechnology, China) and the obtained target gene were ligated overnight at 16°C using T4 DNA Ligase (TaKaRa Biotechnology, China). After the pMD19T-capsid plasmid was transformed into DH5α competent cells, the plasmid DNA was extracted using a Plasmid Mini Kit I (Omega, USA).

### P-S-MCDA Primers and Probes Design

The conserved region of the capsid gene was determined by alignment of PCV3 strains indexed in the GenBank (accession nos: MF589105.1, MF589107.1, MF769811.1, MF769807.1, MF084994.1, KX778720.1, KX898030.1, MG310152.1, MF079254.1, and MG250187.1). According to the principle of S-MCDA, capsid gene sequences of PCV3-US/MO2015 strain (accession no: KX778720.1) were input for P-S-MCDA primer design by using the software Primer Premier 5.0 (17). Among multiple sets of primers, the primers targeting the conserved regions of the capsid gene were selected for subsequent analysis. P-S-MCDA primers used in this study are listed in Table 1. In addition, a probe and its complementary quencher oligonucleotides were included.

**Table 1:** Primers of the P-S-MCDA assay for PCV3.

**Primer name**	**Sequences 5^**′**^ → 3^**′**^**	**Genome position^**a**^**
CP1 (C1+P1)	CTCACCCAGGACAAAGCCTCTT-CATTGAACGGTGGGGTCAT	C1: 1,497–1,518 P1: 1,443–1,461
CP2 (C2+P2)	TGGTTTCGGGGTGAAGTAACGG-AGACGACCCTTATGCGGAA	C2: 1,541–1,562 P2: 1,604–1,622
F1	CCGGGACATAAATGCTCCAA	1,411–1,430
F2	CCACAAACACTTGGCTCCA	1623–1,641
C1	CTCACCCAGGACAAAGCCTCTT	1,497–1,518
D1	CCCACCCCATGGCTCAACA	1,465–1,483
R1	FAM-*^*b*^CGGGTTTGCGCTCAGCCATCCGTTCAGTCCGTCAGGTCAG*-ATTCTGGCGGGAACTACC	1,523–1,540
Quencher	*^*b*^CTGACCTGACGGACTGAACGGATGGCTGAGCGCAAACCCG*-Dabcyl	None

a *The genome of PCV3-US/MO2015 strain (accession no: KX778720.1).*

b *Italic font indicates complementary oligonucleotides flanked at 5′ of R1 primer*.

### P-S-MCDA Assay

To establish the P-S-MCDA, the reaction mixture containing 1 × Isothermal Amplification Buffer (New England Biolabs, USA), 6 mM MgSO_4_ (New England Bio-labs, USA), 1.6 mM High Pure dNTPs (TransGen Biotech, China), and 8U Bst WarmStart DNA Polymerase (New England Biolabs, USA) was prepared. To perform the P-S-MCDA reaction, the concentration of primers was optimized and determined as 1.6 μM CP1/CP2, 0.2 μM F1/F2, 0.8 μM C1/D1, 0.24 μM R1, and 0.32 μM quencher. Then, the reaction tube was incubated in a real-time PCR detection system (Roche Light Cycler 480 II, Switzerland). The reaction program was set as follows: 30 cycles at 60°C for 1 min. FAM fluorescence signals were obtained at the end of each cycle step. The results could be directly judged by color changes or *via* the cycle threshold (Ct) value. A Ct value ≤ 30 indicated positive results, while a Ct value > 30 indicated negative results.

### P-S-MCDA Assay in the Smartphone-Based Cassette

P-S-MCDA is conducted at a constant temperature of 60°C in a water bath for 30 min (YIHENG Technical, China). Then, the reaction tube is placed into a smartphone-based cassette (19), and the photos are obtained using a Nova5z smartphone (Huawei, China). The result of each reaction tube can be determined *via* color judgment by eye. A positive reaction fluoresces green, while a negative reaction has no color.

### TaqMan-Based qPCR

TaqMan-based qPCR of PCV3 was conducted in accordance with the previous report (14). The 25-μL reaction mixture contained 0.4 μM of each primer and probe, 1 × qPCR Probe Master Mix (Vazyme, China), and 2 μL template DNA. The reaction program was set as follows: 95°C for 3 min followed by 40 cycles at 95°C for 10 s and 60°C for 60 s. FAM fluorescence signals were obtained at the end of each annealing step by the real-time PCR detection system (Roche Light Cycler 480 II, Switzerland). Results with a Ct value of <40 were considered positive, while results with no Ct value within 40 cycles were considered negative.

### Specificity Analysis

DNA extracted from PCV1, PCV2, PRV GD-WH strain, HPS, SS, and APP were used as DNA templates for evaluating the specificity of the method. P-S-MCDA and qPCR were conducted in a real-time PCR detection system (Roche Light Cycler 480 II, Switzerland).

### Sensitivity Analysis

The pMD19T-capsid plasmid was diluted to 10^5^, 10^4^, 10^3^, 10^2^, 10^1^, 10^0^, and 10^−1^ copies as standard DNA to assess the sensitivity of P-S-MCDA. The negative control (ddH_2_0) was also tested. The detection limit of the P-S-MCDA was compared with qPCR in parallel.

### Evaluation of Clinical Application

A total of 139 suspected clinical samples and 15 blood samples from SPF pigs were used for DNA extraction and P-S-MCDA detection. Meanwhile, the results were verified by TaqMan-based qPCR.

## Results

### Establishment of the P-S-MCDA

**Figure 1:**
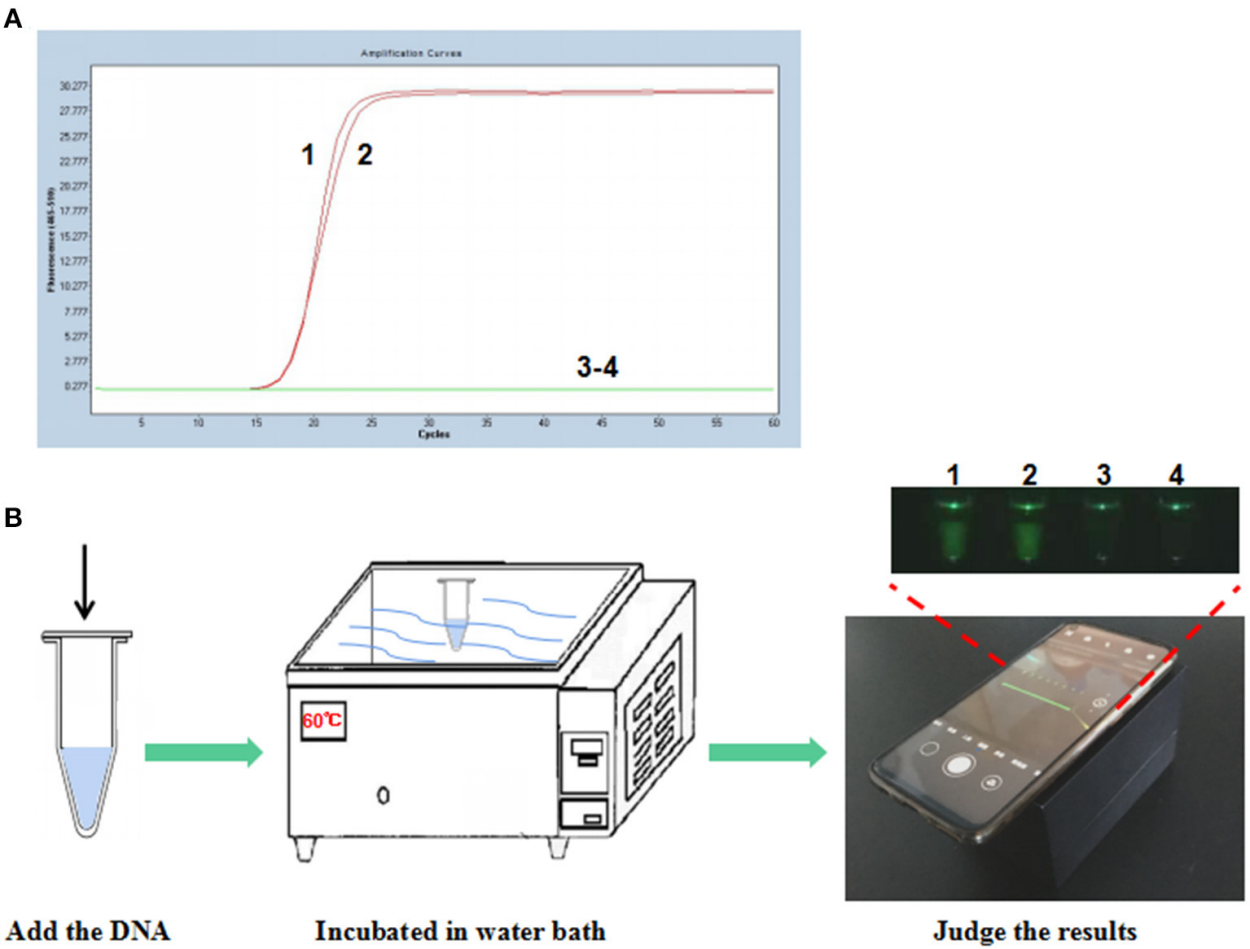
Establishment of the P-S-MCDA assay for PCV3. **(A)** P-S-MCDA method assay by the Roche Light Cycler 480 II. **(B)** Flowchart of the P-S-MCDA method combined with smartphone-based cassette. Lanes or Tubes 1–2, 10^5^ copies of pMD19T-capsid plasmid DNA. Lanes or Tubes 3–4, ddH_2_O.

To establish the P-S-MCDA method, seven primers were used to amplify the target gene. One of the amplified primers, R1, was linked with an extra oligonucleotide and modified at the 5′ end with FAM fluorescence. In the initial reaction system, R1 can be combined by its complementary quencher primer, and the fluorescence signal is dampened. In the process of incubation at 60°C for 30 min, P-S-MCDA primers initiate the circular reaction after binding to the PCV3 capsid gene, and the quencher primer is displaced by the reverse elongation complementary with R1 primer. At this point, a fluorescence signal will be released and monitored. The negative control has no fluorescence curve because no circular reaction exists in the tube. Therefore, the positive result of the P-S-MCDA will be calculated as the Ct value by the real-time PCR detection system or judged by using the smartphone-based cassette (Figures 1A,B).

### Specificity of the P-S-MCDA

**Figure 2:**
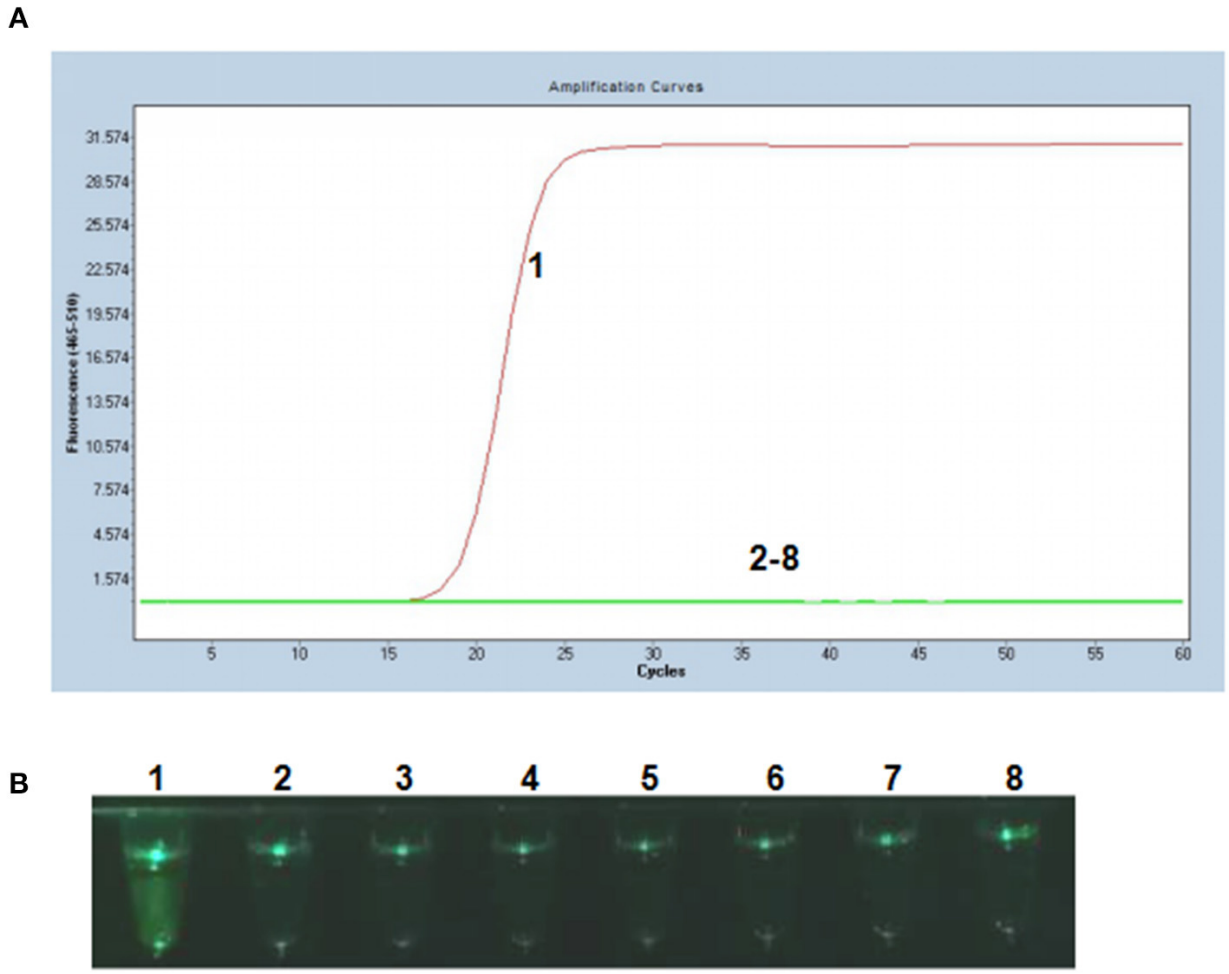
Specificity of the P-S-MCDA assay for PCV3. **(A)** Specificity of P-S-MCDA by the Roche Light Cycler 480 II. **(B)** Specificity of P-S-MCDA by the smartphone-based cassette. Lane or Tube 1, 10^5^ copies of pMD19T-capsid plasmid DNA. Lanes or Tubes 2–8, ddH_2_O, PCV2, PCV1, PRV GD-WH strain, HPS, SS, and APP, respectively.

To evaluate the specificity of the P-S-MCDA method, DNA samples extracted from PCV2, PCV1, PRV GD-WH strain, HPS, SS, and APP were analyzed in parallel with the PCV3 capsid gene. The results showed that only the PCV3 capsid gene could initiate the P-S-MCDA reaction with a fluorescence curve (Figure 2A) or a fluoresces green tube (Figure 2B). This demonstrated the specificity of the P-S-MCDA.

### Sensitivity of the P-S-MCDA

**Figure 3:**
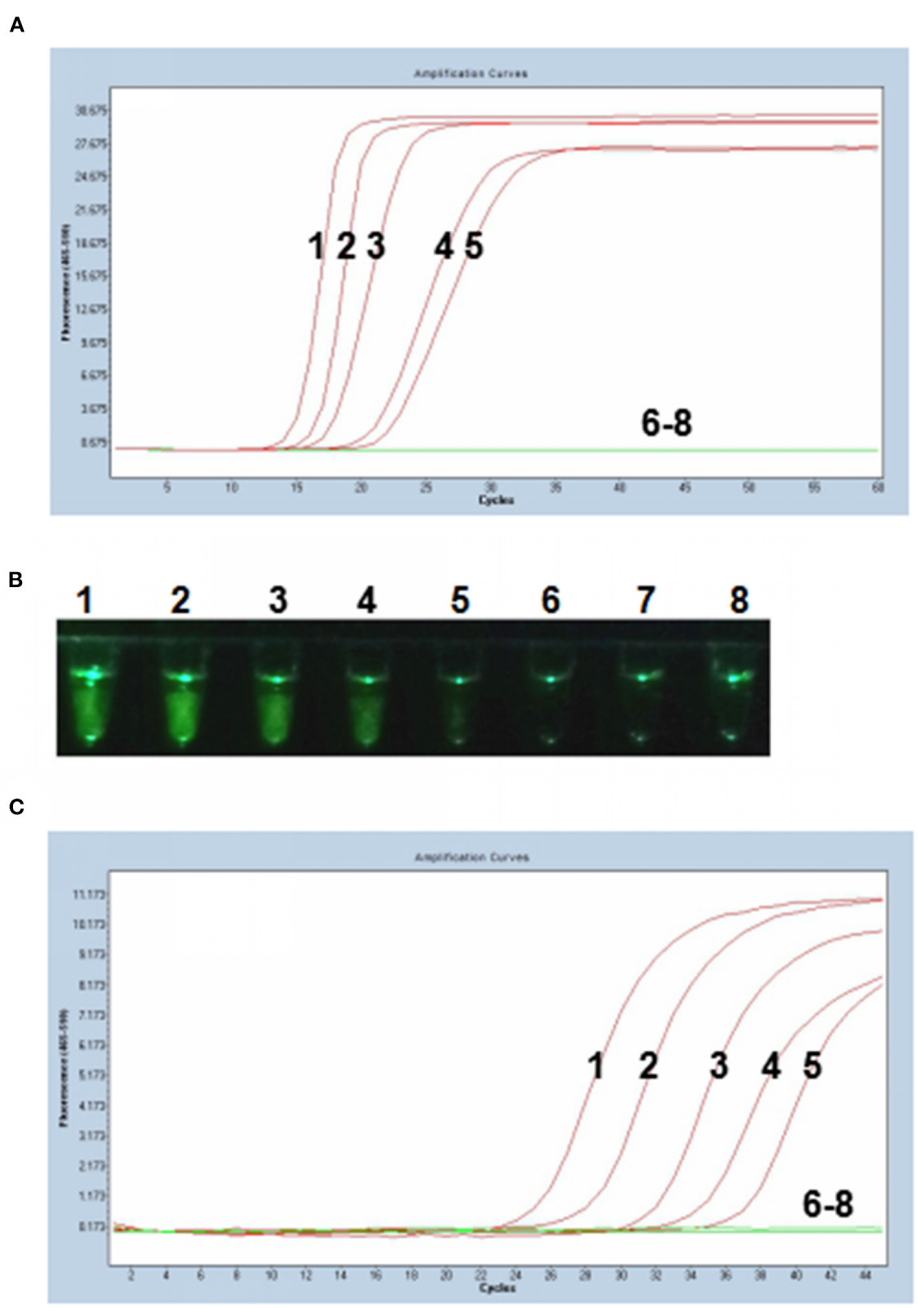
Comparison of sensitivity of the P-S-MCDA and qPCR. **(A)** Sensitivity of P-S-MCDA by the Roche Light Cycler 480 II. **(B)** Sensitivity of P-S-MCDA using a smartphone-based cassette. **(C)** Sensitivity of qPCR by the Roche Light Cycler 480 II. Lanes or Tubes 1–7, DNA of pMD19T-capsid plasmid (10^5^, 10^4^, 10^3^, 10^2^, 10^1^, 10^0^, and 10^−1^ copies). Lane or Tube 8, ddH_2_O.

Tenfold serial dilutions of PCV3 capsid gene DNA (10^5^, 10^4^, 10^3^, 10^2^, 10^1^, 10^0^, and 10^−1^ copies) were used as DNA templates to compare the detection limits of the P-S-MCDA with qPCR. In this study, P-S-MCDA displayed a minimum detection limit of 10 copies in 30 min (Figure 3A). Moreover, we found that the fluorescence signal was strong enough to be observed in a smartphone-based cassette (Figure 3B). When the serially diluted samples were analyzed by qPCR, the 10 copies detection limit was also observed (Figure 3C).

### Evaluation of Clinical Application

Among 139 clinical samples, 40 positive results were detected by the qPCR method. The positive rate was 28.78% (40/139). Additionally, the P-S-MCDA assay showed a consistent positive rate with TaqMan-based qPCR (Table 2). Furthermore, 15 blood tissues sampled from SPF pigs were analyzed by using both P-S-MCDA and qPCR to test the accuracy of P-S-MCDA for diagnosis of negative animals. Negative results were obtained using both methods.

**Table 2:** Detection of PCV3 in clinical samples by the P-S-MCDA and qPCR.

**Method**	**No. of positive**	**No. of negative**	**Detection**
	**samples**	**samples**	**rate^**a**^**
P-S-MCDA	40	99	28.78%
qPCR	40	99	28.78%

a *Detection rate was defined by no. of positive samples/no. of total samples(%)*.

## Discussion

Since PCV3 was identified in 2016, further studies have uncovered its pathological features in piglets and cross-species transmission possibility. Therefore, it is necessary to establish a highly efficient and sensitive detection method to perform further molecular epidemiological investigations and facilitate timely control. In this study, we established a novel PCV3 P-S-MCDA detection method.

The P-S-MCDA combines the advantages of qPCR and isothermal strand-displacement polymerization reaction in that the method not only ensures the high sensitivity of qPCR but also can amplify target genes under constant temperature. Compared with qPCR, the reaction without complex thermal deformation steps only needs to be in a constant temperature environment of 60°C, so it can be easily performed. Compared with colorimetric isothermal amplification, P-S-MCDA is a probe-based real-time fluorescence detection method, avoiding the possible non-specific reactions and the uncertainty of results caused by visual errors. Moreover, the reaction system can be carried out at a constant temperature of 60°C and completed within 30 min, and the results can be visually judged by the real-time detection system or using a smartphone-based cassette (19). The test can save 20 min compared with novel polymerase spiral reaction (20), 40 min compared with real-time loop-mediated isothermal amplification (15), and at least 60 min compared with qPCR or PCR (13, 14).

The PCV3 ORF2 gene is associated with host-range specificity and virus independence as it encodes the capsid protein of PCV3. The gene has a strongly conserved sequence. Therefore, it is advisable to use ORF2 as a target fragment for nucleic acid detection (21, 22). In our study, seven primers of the P-S-MCDA spanning eight distinct regions of the target gene were designed using the software Primer Premier 5.0. The primer design procedure is easier to perform than LAMP. The analysis showed that this method had good specificity, with no cross false reaction with other porcine viral pathogens. Most isothermal amplification techniques use color change (4, 20) or turbidity (17) to interpret the results, but P-S-MCDA monitors the amplification of the target gene in real time quantitatively by the real-time PCR detection system. Therefore, the P-S-MCDA not only avoids the error of visual interpretation of the results but also is capable of quantitative analysis of target gene sequences. The detection limit of the P-S-MCDA is as low as 10 copies, consistent with qPCR and 10 times more sensitive than previously reported PCR results (18, 23–25). Moreover, a practical pathogen detection method not only needs to be rapid, specific, sensitive, and simple but also to be economical. Compared to many testing methods, P-S-MCDA has desirable economical features such as lower reagent cost compared to RAA (26) and less equipment requirements than droplet digital PCR (27).

In analyzing clinical samples, P-S-MCDA showed a consistent accuracy rate compared with qPCR methods. What is noticeable is that some laboratories on the clinical frontier may have limit advanced equipment, such as the expensive real-time PCR detection system. Moreover, another advance of the P-S-MCDA method is that it can be combined with a smartphone-based cassette, and thus only a simple box can be used to determine the results by eye. Therefore, the P-S-MCDA can be used to real-time quantitative monitor of target genes by the real-time PCR detection system and can also be applied in rural areas with limited facilities.

In summary, the P-S-MCDA assay was demonstrated to be a simple, rapid, sensitive, specific, and economical detection method for PCV3. It is valuable for PCV3 real-time detection in laboratories or at point of care testing in rural areas.

## Data Availability Statement

The raw data supporting the conclusions of this article will be made available by the authors, without undue reservation.

## Ethics Statement

The animal study was reviewed and approved by Institute of Animal Health, Guangdong Academy of Agricultural Sciences, China. The license number was SYXK (Yue) 2011-0116.

## Author Contributions

ZB performed the experiments and drafted the manuscript. HG conceived the experiments and revised the manuscript. PC, KZ, and DY prepared materials for the experiments. SS, ZJ, and YL participated in the analysis of the data. CL and RC supervised the study. All authors read and approved the final manuscript.

## Conflict of Interest

The authors declare that the research was conducted in the absence of any commercial or financial relationships that could be construed as a potential conflict of interest.

## Publisher's Note

All claims expressed in this article are solely those of the authors and do not necessarily represent those of their affiliated organizations, or those of the publisher, the editors and the reviewers. Any product that may be evaluated in this article, or claim that may be made by its manufacturer, is not guaranteed or endorsed by the publisher.

## References

[1] Han L Yuan GF Chen SJ Dai F Hou LS Fan JH. Porcine circovirus type 2 (PCV2) infection in Hebei Province from 2016 to 2019: a retrospective study. Arch Virol. (2021) 166:2159–2171. 10.1007/s00705-021-05085-z 34031716

[2] Collins PJ McKillen J Allan G. Porcine circovirus type 3 in the UK. Vet Rec. (2017) 181:599. 10.1136/vr.j5505 29192047

[3] Fux R Söckler C Link EK Renken C Krejci R Sutter G. Full genome characterization of porcine circovirus type 3 isolates reveals the existence of two distinct groups of virus strains. Virol J. (2018) 15:25. 10.1186/s12985-018-0929-3 29378597 PMC5789634

[4] Gou H Bian Z Cai R Jiang Z Song S Li Y. The colorimetric isothermal multiple-self-matching-initiated amplification using cresol red for rapid and sensitive detection of porcine circovirus 3. Front Vet Sci. (2020) 7:407. 10.3389/fvets.2020.00407 32851005 PMC7417626

[5] Hayashi S Ohshima Y Furuya Y Nagao A Oroku K Tsutsumi N. First detection of porcine circovirus type 3 in Japan. J Vet Med Sci. (2018) 80:1468–72. 10.1292/jvms.18-0079 30078831 PMC6160883

[6] Tochetto C Lima DA Varela APM Loiko MR Paim WP Scheffer CM. Full-genome sequence of porcine circovirus type 3 recovered from serum of sows with stillbirths in Brazil. Transbound Emerg Dis. (2018) 65:5–9. 10.1111/tbed.12735 29027372

[7] Yuzhakov AG Raev SA Alekseev KP Grebennikova TV Verkhovsky OA Zaberezhny AD. First detection and full genome sequence of porcine circovirus type 3 in Russia. Virus Genes. (2018) 54:608–11. 10.1007/s11262-018-1582-z 29948781

[8] Jiang H Wang D Wang J Zhu S She R Ren X. Induction of porcine dermatitis and nephropathy syndrome in piglets by infection with porcine circovirus type 3. J Virol. (2019) 93:e02045–18. 10.1128/jvi.02045-18 30487279 PMC6363995

[9] Ouyang T Niu G Liu X Zhang X Zhang Y Ren L. Recent progress on porcine circovirus type 3. Infect Genet Evol. (2019) 73:227–33. 10.1016/j.meegid.2019.05.009 31096019

[10] Jiang H Wei L Wang D Wang J Zhu S She R. ITRAQ-based quantitative proteomics reveals the first proteome profiles of piglets infected with porcine circovirus type 3. J Proteomics. (2020) 212:103598. 10.1016/j.jprot.2019.103598 31785380

[11] Liu Y Meng H Shi L Li L. Sensitive detection of porcine circovirus 3 by droplet digital PCR. J Vet Diagn Invest. (2019) 31:604–7. 10.1177/1040638719847686 31046639 PMC6857016

[12] Nguyen NH Do Tien D Nguyen TQ Nguyen TT Nguyen MN. Identification and whole-genome characterization of a novel Porcine Circovirus 3 subtype b strain from swine populations in Vietnam. Virus Genes. (2021) 57:385–389. 10.1007/s11262-021-01844-x 33993380

[13] Hou CY Xu T Zhang LH Cui JT Zhang YH Li XS. Simultaneous detection and differentiation of porcine circovirus 3 and 4 using a SYBR Green ı-based duplex quantitative PCR assay. J Virol Methods. (2021) 293:114152. 10.1016/j.jviromet.2021.114152 33845107

[14] Palinski R Piñeyro P Shang P Yuan F Guo R Fang Y. A novel porcine circovirus distantly related to known circoviruses is associated with porcine dermatitis and nephropathy syndrome and reproductive failure. J Virol. (2017) 91:e01879–16. 10.1128/jvi.01879-16 27795441 PMC5165205

[15] Wang H Liu X Zeng F Zhang T Lian Y Wu M. Development of a real-time loop-mediated isothermal amplification assay for detection of porcine circovirus 3. BMC Vet Res. (2019) 15:305. 10.1186/s12917-019-2037-z 31443656 PMC6706899

[16] Wang J Zhang Y Zhang R Han Q Wang J Liu L. Recombinase polymerase amplification assay for rapid detection of porcine circovirus 3. Mol Cell Probes. (2017) 36:58–61. 10.1016/j.mcp.2017.09.001 28958719

[17] Wang Y Wang Y Ma AJ Li DX Luo LJ Liu DX. Rapid and sensitive isothermal detection of nucleic-acid sequence by multiple cross displacement amplification. Sci Rep. (2015) 5:11902. 10.1038/srep11902 26154567 PMC4648395

[18] Ku X Chen F Li P Wang Y Yu X Fan S. Identification and genetic characterization of porcine circovirus type 3 in China. Transbound Emerg Dis. (2017) 64:703–8. 10.1111/tbed.12638 28317326 PMC7169768

[19] Wen J Gou H Wang S Lin Q Chen K Wu Y. Competitive activation cross amplification combined with smartphone-based quantification for point-of-care detection of single nucleotide polymorphism. Biosens Bioelectron. (2021) 183:113200. 10.1016/j.bios.2021.113200 33819904

[20] Ji J Xu X Wang X Zuo K Li Z Leng C. Novel polymerase spiral reaction assay for the visible molecular detection of porcine circovirus type 3. BMC Vet Res. (2019) 15:322. 10.1186/s12917-019-2072-9 31492192 PMC6731610

[21] Oh T Chae C. First isolation and genetic characterization of porcine circovirus type 3 using primary porcine kidney cells. Vet Microbiol. (2020) 241:108576. 10.1016/j.vetmic.2020.108576 31928694

[22] Chung HC Nguyen VG Park YH Park BK. Genotyping of PCV3 based on reassembled viral gene sequences. Vet Med Sci. (2021) 7:474–82. 10.1002/vms3.374 33040453 PMC8025635

[23] Wang J Zhang Y Wang J Liu L Pang X Yuan W. Development of a TaqMan-based real-time PCR assay for the specific detection of porcine circovirus 3. J Virol Methods. (2017) 248:177–80. 10.1016/j.jviromet.2017.07.007 28743583

[24] Yang K Jiao Z Zhou D Guo R Duan Z Tian Y. Development of a multiplex PCR to detect and discriminate porcine circoviruses in clinical specimens. BMC Infect Dis. (2019) 19:778. 10.1186/s12879-019-4398-0 31488066 PMC6727504

[25] Yuan L Liu Y Chen Y Gu X Dong H Zhang S. Optimized real-time fluorescence PCR assay for the detection of porcine Circovirus type 3 (PCV3). BMC Vet Res. (2020) 16:249. 10.1186/s12917-020-02435-y 32680512 PMC7368764

[26] Li Y Yu Z Jiao S Liu Y Ni H Wang Y. Development of a recombinase-aided amplification assay for rapid and sensitive detection of porcine circovirus 3. J Virol Methods. (2020) 282:113904. 10.1016/j.jviromet.2020.113904 32470487

[27] Zhang Y Zhang Z Wang Z Wang Z Wang C Feng C. Development of a droplet digital PCR assay for sensitive detection of porcine circovirus 3. Mol Cell Probes. (2019) 43:50–7. 10.1016/j.mcp.2018.11.005 30468765

